# Evidence That Speciation of Oxovanadium Complexes Does Not Solely Account for Inhibition of *Leishmania* Acid Phosphatases

**DOI:** 10.3389/fchem.2018.00109

**Published:** 2018-04-12

**Authors:** Benjamin M. Dorsey, Craig C. McLauchlan, Marjorie A. Jones

**Affiliations:** Department of Chemistry, Illinois State University, Normal, IL, United States

**Keywords:** oxovanadium, decavanadate, phosphatase inhibition, Leishmania, acid phosphatase, enzyme studies

## Abstract

Leishmaniasis is an endemic disease affecting a diverse spectra of populations, with 1.6 million new cases reported each year. Current treatment options are costly and have harsh side effects. New therapeutic options that have been previously identified, but still underappreciated as potential pharmaceutical targets, are *Leishmania* secreted acid phosphatases (SAP). These acid phosphatases, which are reported to play a role in the survival of the parasite in the sand fly vector, and in homing to the host macrophage, are inhibited by orthovanadate and decavanadate. Here, we use *L. tarentolae* to further evaluate these inhibitors. Using enzyme assays, and UV-visible spectroscopy, we investigate which oxovanadium starting material (orthovanadate or decavanadate) is a better inhibitor of *L. tarentolae* secreted acid phosphatase activity *in vitro* at the same total moles of vanadium. Considering speciation and total vanadium concentration, decavanadate is a consistently better inhibitor of SAP in our conditions, especially at low substrate:inhibitor ratios.

## Introduction

### *Leishmania*, leishmaniasis, and current treatment options

Leishmaniasis is defined by the Center for Disease Control and Prevention as a neglected tropical disease carried by the sand fly vector. It affects populations in Asia, India, the Middle East, Africa, Central and South America, and southern Europe. This disease is caused by any of 20 species of the parasitic protozoan *Leishmania* (CCDC, 2013b). Leishmaniasis presents clinically in three forms: visceral, cutaneous, and mucocutaneous (CCDC, 2013b). Over 1.6 million new cases of leishmaniasis are reported yearly (CCDC, 2013b), and current treatment options include: pentavalent antimony salts, amphotericin B, liposomal amphotericin B, ketoconazole, itraconazole, and fluconazole (CCDC, 2013a). Treatments can cost from $ 20 to $252 USD per day, and treatments can last from 20 days to 4 months or longer depending on how long it takes for the lesion to heal (Monzote, 2009; CCDC, 2013b). *Leishmania* diseases are becoming more wide spread, and there are few good drug therapies, thus new directions of treatments should be explored.

### Polyoxometalates

Polyoxometalates (POM) have been known for many years (Wu, 1920; Dawson, 1953; Pope, 1976; Acerete et al., 1979a,b) with a range of interesting uses (Pope and Müller, 1991). Their use in medicine has also been investigated (Hill et al., 1990a,b; Rhule et al., 1998) and the interaction with proteins, amino acids, and DNA have been examined (Steens et al., 2010; Goovaerts et al., 2013; Arefian et al., 2017). Of particular interest to this manuscript (*vide infra*), is decavanadate (V_10_, V_10_${\text{O}}_{28}^{6-}$), which has gained recent interest for its biological activity (Aureliano and Gândara, 2005; Aureliano and Crans, 2009; Turner et al., 2012; Aureliano, 2016).

### Phosphatases

Phosphatases are hydrolytic enzymes (EC 3.1) that are responsible for the hydrolysis of phosphoesters from substrate producing a phosphate and an alcohol (Bairoch, 2000). Classically, there are three general types of phosphatases; acid phosphatases, neutral phosphatases, and alkaline phosphatases (Vincent et al., 1992; Gani and Wilkie, 1997). These phosphatases are categorized based upon their pH optimum, although alternative classification systems do exist. Most relevant to this work are acid phosphatases. Acid phosphatases (AP) are located, in humans, in the cellular components of bone, spleen, kidney, liver, intestine, and are also found in the blood (Henneberry et al., 1979; Anand and Srivastava, 2012). *Leishmania* have been reported by a number of other laboratories to contain two different genes for secreted acid phosphatases (SAP) (Gottlieb and Dwyer, 1982; Ilg et al., 1994; Fernandes et al., 2013). The pathogenesis of *Leishmania* changes during the life cycle from the amastigote form to the promastigote form of the parasite (CCDC, 2013b). *In vitro* parasites in the stationary phase of their growth curve are more infective to macrophages than are parasites in the logarithmic phase (Mojtahedi et al., 2008; Navabi and Soleimanifard, 2015). It has also been reported that the kinetic parameters of SAP isolated from the *in vitro* stationary phase of *Leishmania major* change, such that the enzymes have a larger V_max_ and a smaller K_m_ compared to the logarithmic phase enzyme (Fernandes et al., 2013). *Leishmania* SAP are established to play several roles during the life cycle of the parasite, including: aiding in the survival of the parasite in the sand fly alternative host (Baghaei and Mesripour, 2003; Fernandes et al., 2013), and formation of the parasitophorous vacuole, thus preventing macrophages from forming hydrogen peroxide (Baghaei and Mesripour, 2003). Thus, *Leishmania* SAP are of interest as potential pharmaceutical targets for the treatment of leishmaniasis.

### *L. tarentolae* secreted acid phosphatase enzymes

*Leishmania tarentolae* serves as a good model system for the investigation of the two *Leishmania* SAP for several reasons. First, *L. tarentolae* are easy to grow, and their growth in culture is easily assessed (Morgenthaler et al., 2008). *L. tarentolae* do not infect humans, thus lowering the risk for investigators. *L. tarentolae* have utility in the macrophage model system used to assess infectivity. Finally, *L. tarentolae* are sensitive to current treatment options, thus therapeutics that are effective in this model system may also be potentially effective for work the human parasite, *L. major* (Taylor et al., 2010).

**Figure 1:**
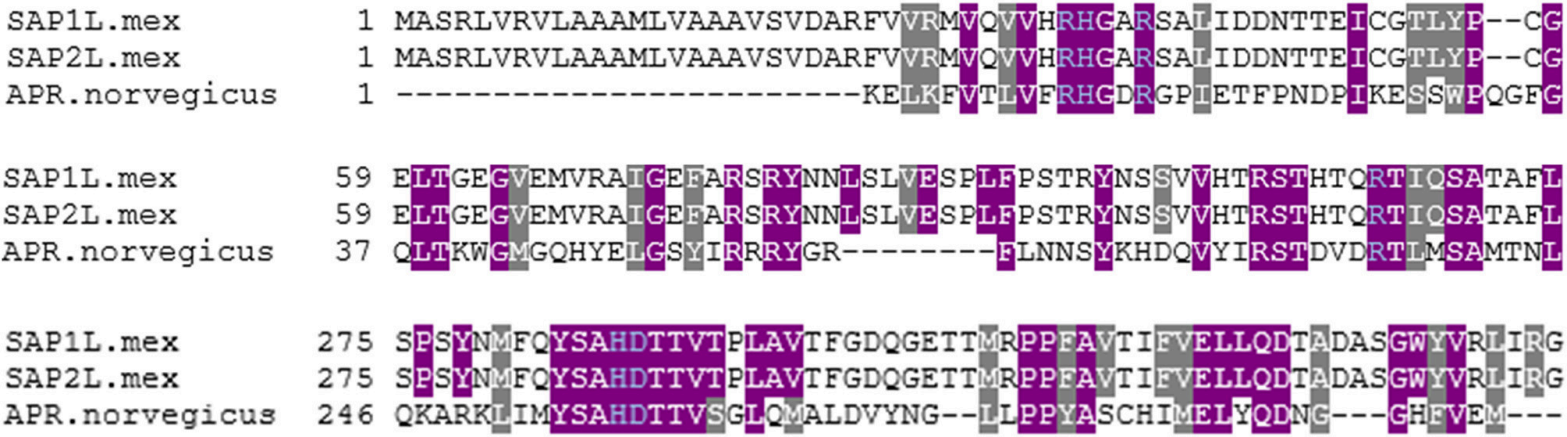
Multiple sequence alignment of three acid phosphatases. This sequence alignment was completed using Kalign (Lassmann and Sonnhammer, 2005) and the BoxShade Server (Hoffmann and Baron, 2014). Gray highlights indicate different, but similar, amino acids between species. Purple highlights indicate identical amino acids between species. Blue text indicates likely residues for coordinating vanadate.

Using acid phosphatase amino acid sequence alignment from rat (*Rattus norvegicus* EC3.1.3.2, PDB 1rpt) as a comparative model to *Leishmania* SAP, it can be seen that there is overlap between the reported vanadate binding residues in the *R. norvegicus* and the *L. mexicana* acid phosphatases (SAP1L.mex gene accession number Z46969.1 and SAP2L.mex gene accession number Z46970.1) as shown in Figure 1. The gray and purple highlights are the amino acids reported to be in the active site of these acid phosphatases. Gray highlights indicate different, but similar, amino acids between species. Purple highlights indicate identical amino acids between species. Using the amino acid numbering from *R. norvegicus*, the following residues (Arg11, His12, Arg15, Arg79, His257, and Asp258 is also sometimes indicated; Lindqvist et al., 1994) are responsible for coordinating vanadate, and are highlighted in blue in Figure 1. In the structure of AP from *R. norvegicus* (Lindqvist et al., 1994), His12 directly coordinates to vanadium, whereas the other residues indicated are all involved in secondary interactions.

### Vanadium background, vanadium human exposure, vanadium chemistry, and speciation

**Figure 2:**
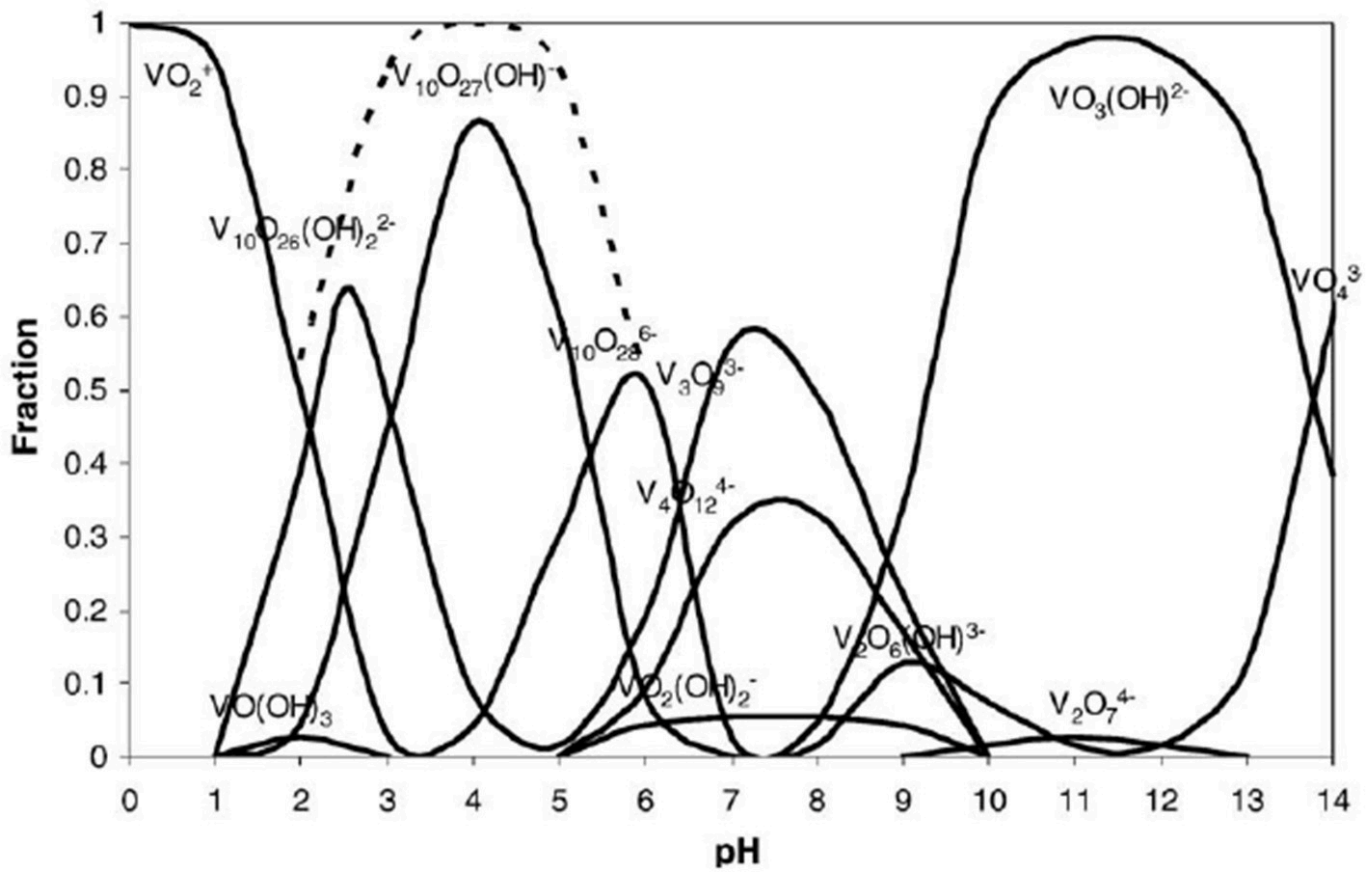
Vanadium (V) speciation as a mole fraction of total vanadium present at a given pH, over the typical pH scale (0–14). This speciation diagram is for a 0.1 molal vanadium solution. Reproduced from Baes and Mesmer (1976) with permission from Wiley and Sons.

The typical total amount of vanadium in humans is about 1 mg (Rehder, 2013). Vanadium is a very versatile element and has five common oxidation states available, with the vanadium (V) oxidation state being the overwhelmingly dominant species in aqueous solution under typical pH and reduction potential conditions (Baes and Mesmer, 1976; Crans et al., 2004). Vanadium forms covalent adducts with oxygen, which produce numerous different species of vanadium in its (V) oxidation state as a function of pH (Figure 2), including a broad variety of polyoxometalates (POMs; Baes and Mesmer, 1976). This speciation is of critical importance when examining aqueous vanadium solutions (Levina et al., 2017).

**Figure 3:**
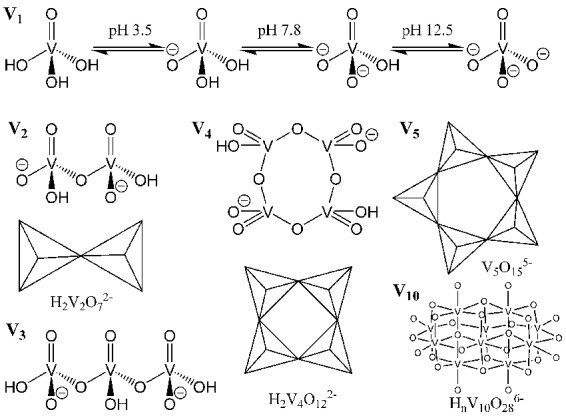
Representation of common vanadium polyoxometalates in aqueous solution. Reproduced with permission from McLauchlan et al. (2015) with permission from Elsevier.

Speciation of vanadium, specifically decavanadate speciation in acidic media, has been investigated (Figures 2, 3; Baes and Mesmer, 1976). It is clear that under acidic conditions, protonation status changes either by deprotonation or cation exchange with the medium, and vanadium speciation is a function of vanadium concentration, solution pH, and ionic strength of the solution (Rossotti and Rossotti, 1956; Corigliano and Di Pasquale, 1975; Baes and Mesmer, 1976; Crans et al., 2004). Because of this, vanadium speciation, degree of protonation, and degree of proton displacement by cations in solution are likely different for solutions of different composition. Therefore, when using decavanadate or orthovanadate as inhibitors of phosphatases under assay conditions (often involving various buffers and cell growth media), it is not always clear what species are present, or what species are responsible for inhibition of the enzyme being assayed.

### Vanadium is a potential medicinal agent as a phosphatase inhibitor

It is well known that oxovanadium species act as phosphatase inhibitors with varying efficacy (Van Etten et al., 1974; Abbott et al., 1979; Knowles, 1980; Gresser et al., 1987; Gordon, 1991; Li et al., 2008; Crans, 2015; McLauchlan et al., 2015). How vanadium acts as a phosphatase inhibitor is thought to be through the action of a vanadium (V) monomeric oxyanion, vanadate, mimicking a five-coordinate high energy intermediate of the transition state phosphate, therefore behaving as a competitive inhibitor (Van Etten et al., 1974; Abbott et al., 1979; Knowles, 1980; Gresser et al., 1987; Gordon, 1991; Li et al., 2008; Crans, 2015; McLauchlan et al., 2015). There are numerous crystal structures of phosphatases, with nitrogen-containing, oxygen containing, or sulfur-containing active site amino acids deposited in the Protein Data Bank (Berman et al., 2000), that have been recently reviewed (Crans, 2015; McLauchlan et al., 2015). These phosphatases function to hydrolyze esters, phosphoesters, and phosphoanhydrides. These crystal structures had been determined from crystals soaked with vanadium complexes; the majority of experiments using orthovanadate, at pH values ranging from 5.40 to 8.00. The overwhelming majority of these phosphatases, regardless of the type of active site amino acid residues (O-, N-, or S-containing), have a monomeric form of vanadium (${\text{VO}}_{3}^{1-}$ or ${\text{VO}}_{4}^{3-}$) present in the phosphatase active site upon solving the crystal structure. It should be noted at the employed concentrations of vanadium and in this pH range, that di-, tri-, and tetrameric vanadium species V_2_, V_3_, and V_4_ (Figure 3), respectively, are predicted (Baes and Mesmer, 1976; Rigden et al., 2003; Crans, 2015; McLauchlan et al., 2015) to be the major forms of vanadium present, and not the monomeric form(s) that are reported in the enzyme's active site. Thus, there is a discrepancy between what one might hypothesize about the species responsible for inhibition (the major species present may be responsible for the inhibition), and what one actually finds (a minor species present may be responsible for the inhibition). There may also be speciation ambiguities under experimental conditions using solutions of a complex nature that are not easily resolved by current predictors of vanadium speciation. Regardless, it is thought that these monomeric species are likely responsible for phosphatase inhibition. There is, however, a likely discrepancy between solid state speciation of vanadium that occurs under soaking conditions, and aqueous speciation of vanadium that occurs in enzymatic assays because crystal dynamic conditions are likely to be different than those of the more flexible protein under enzymatic assay conditions in terms of pH, ionic strength, and vanadium concentration. Therefore, the species of vanadium present in enzymatic assays cannot necessarily be assumed to be the same as the species present after crystal soaking experiments. To further stress the importance of speciation, crystallographic soaking studies that used metavanadate (50 mM) starting material, under acidic conditions (pH 5.0) produced protein crystals of *Bacillus stearothermophilus* phosphatase with trivanadate, V_3_${\text{O}}_{8}^{2-}$ (V_3_), located in the putative active site, when the authors had expected to find orthovanadate, ${\text{VO}}_{4}^{3-}$ (PDB ID 1h2f.) (Rigden et al., 2003).

We have previously reported that a number of vanadium compounds (including orthovanadate, decavanadate, and complexes with picolinate or imidazolyl-carboxylate as ligands) negatively affect *L. tarentolae* viability as well as secreted acid phosphatase activity *in vitro* (Turner et al., 2012; Mendez et al., 2014). However, in those studies we did not address the potential problem of speciation. To further investigate the ambiguities of vanadium speciation and phosphatase inhibition, we used a previously published model (Baumhardt et al., 2015) involving plotting the log of substrate divided by inhibitor effects on product formation; this model was demonstrated to be useful for comparing competitive enzyme inhibitors. Thus, here we use the model to compare decavanadate (V_10_${\text{O}}_{28}^{6-}$, V_10_) and orthovanadate (${\text{VO}}_{4}^{3-}$, V_1_) as *in vitro* inhibitors of *L. tarentolae* SAP. Several studies of anti-trypanosomal (Urquiola et al., 2006; Benítez et al., 2009; Gambino, 2011; Demoro et al., 2012; Fernández et al., 2013) and anti-leishmanial (Turner et al., 2012; Adriazola et al., 2014; Mendez et al., 2014; Machado et al., 2015; Christensen et al., 2016) activity of vanadium complexes have been reported. The current studies can give insight into the clinical use of these and other vanadate complexes as anti-*Leishmania* therapies.

## Experimental

### Materials and methods

#### Cell culture of *L. tarentolae* and assessment of cell viability by the MTT viability assay

*L. tarentolae* (ATCC 30143) promastigote cells were sterilely grown in brain heart infusion medium (BHI; 37.0 g/L) supplemented with hemin (10 μM), penicillin (10,000 units/mL), and streptomycin (10 mg/mL) following the methods of Morgenthaler et al. (2008). *L. tarentolae* cell viability was assessed by the 3-(4,5-dimethylthiazol-2-yl)-2,5-diphenyltetrazolium bromide (MTT) viability assay (Mosmann, 1983). The MTT assay serves as a quantitative measure (A595 nm) of cell mitochondrial activity, and therefore indirectly monitors cell viability. Sample absorbance at A595 nm was determined with an iMark microplate reader (BioRad Laboratories, Hercules, CA). The BHI growth medium alone was considered as a blank value subtracted from the sample absorbance (BHI and cells). Results are reported as corrected absorbance (A595 nm/Hr incubation with MTT reagent, or A595 nm/Hr incubation with medium only) mean ± standard deviation (*SD*) for *n* = 4 replicates. In this work, the parasites were grown at room temperature in 25 cm^2^ canted flasks (Corning, Inc.; Product number 430372). Samples for assessment by MTT assay were collected daily using sterile technique.

#### Preparation of the *L. tarentolae* acid phosphatase enzyme source

A sample of *L. tarentolae* from each stage of the growth curve (lag, log, stationary, and senescence) was collected and centrifuged (2,000 × g, 10°C, 10 min). The supernatant was collected and stored on ice until it was used for acid phosphatase enzyme assays.

#### Secreted acid phosphatase enzyme assay

SAP activity was evaluated using *para*-nitrophenyl phosphate (*p*NPP, Sigma Aldrich) as substrate following the method of Mendez et al. (2014). This assay at room temperature was performed in 1.5 mL polypropylene tubes in a total reaction volume of 0.9 mL. Sodium acetate buffer (500 μL, 0.5 M, pH 4.5) was used. The enzyme source was *L. tarentolae* cell supernatant from the log phase of the growth curve (300 μL). Then 100 μL substrate (5 mg *p*NPP/1 mL buffer; 20 mM), made in sodium acetate buffer (0.5 M, pH 4.5) was added to start the reaction. After room temperature incubation for 23 h (under apparent first order conditions), the reaction was stopped with addition of 100 μL of 10 M sodium hydroxide, and samples were vortexed. Product formation was measured by spectroscopy at A405 nm. BHI was used to replace enzyme source for spectrophotometric blanks. Data are reported as corrected absorbance (A405 nm) per day in culture. Product (*para-*nitrophenolate) was calculated from corrected A405 nm/23 h by dividing by molar absorptivity (18,000 cm^−1*^ M^−1^) and reported as μM/ 23 hr.

#### Secreted acid phosphatase enzyme inhibition assay

To determine which form of vanadium is a better inhibitor, decavanadate, or orthovanadate, of *L. tarentolae* secreted acid phosphatase enzyme activity the method of Baumhardt et al. (2015) was used. Using the previously determined k_M_ substrate concentration of 391 μM (Mendez et al., 2014), the log ratio of substrate to total vanadium concentration in the assay was calculated for either orthovanadate or decavanadate (as shown in Table 1).

**Table 1:** The concentrations of vanadium (I) used as either total vanadium, or orthovanadate, or decavanadate in each sample.

**Sample**	**log[S]/[I]**	**[Total Vanadium] μM**	**[Decavanadate] μM**	**[Orthovanadate] μM**
1	−2.0	39,100	3,910	39,100
2	−1.5	12,400	1,240	12,400
3	−1.0	3,910	391	3,910
4	−0.5	1,240	124	1,240
5	0.0	391	39.1	391
6	0.5	124	12.4	124
7	1.0	39.1	3.91	39.1
8	1.5	12.4	1.24	12.4
9	2.0	3.91	0.391	3.91

*Substrate was constant at 391 μM in all assays*.

Table 1 indicates the relationship between the log of substrate to inhibitor ratio to the total moles of vanadium, or the total moles of decavanadate, or the total moles of orthovanadate in the assay. It should be noted that for every mole of orthovanadate, there is one mole of vanadium. For every mole of decavanadate, there are 10 moles of vanadium. Thus, orthovanadate was used at 10 times the molar concentration of decavanadate, but the total moles of vanadium from either compound, in the assay, was the same as listed in Table 1.

The order of addition of material to the assay was as follows: Sodium acetate buffer (0.5 M, pH 4.5 from Fisher Scientific), vanadium as either sodium orthovanadate (Acros Organics) or ammonium decavanadate (as synthesized by Turner et al., 2012) freshly prepared in assay buffer, and enzyme source (300 μL of log phase *L. tarentolae* cell supernatant) were added to the assay and allowed to preincubate at room temperature for 10 min. Following substrate addition, assays were incubated at room temperature for 23 h. To stop the reaction, sodium hydroxide (100 μL,10 M) was added and the samples were vortexed. Product was evaluated by spectroscopy at A405 nm. Spectrophotometric blanks were prepared using the same volumes of assay buffer, vanadium as either orthovanadate or decavanadate, and substrate as experimental samples. The enzyme source was replaced with brain heart infusion, the same medium the enzyme was in for kinetic and inhibition assays. Data are reported as mean ± standard deviation for n = 4 replicates.

#### UV-visible comparison of decavanadate to orthovanadate

To assess if samples containing starting material decavanadate or starting material orthovanadate (same total vanadium concentration between samples being compared) are different by electronic absorption (UV-Visible) spectroscopy, samples were prepared (*n* = 3 replicates). The sample contents were identical to those used in the Baumhardt et al. (2015) inhibition studies (Table 1). However, enzyme source was replaced with BHI medium, and substrate was replaced with an equal volume of assay buffer. Samples were prepared, allowed to rest for 23 h, and then samples were evaluated by spectroscopy. Local maxima that were consistently present, but whose amplitude changed with changes in total vanadium concentration, between all samples were identified and used to compare samples containing the same total vanadium concentration either as decavanadate starting material or orthovanadate starting material.

## Results and discussion

### Cell culture of *L. tarentolae* and assessment of cell viability by the MTT viability assay

**Figure 4:**
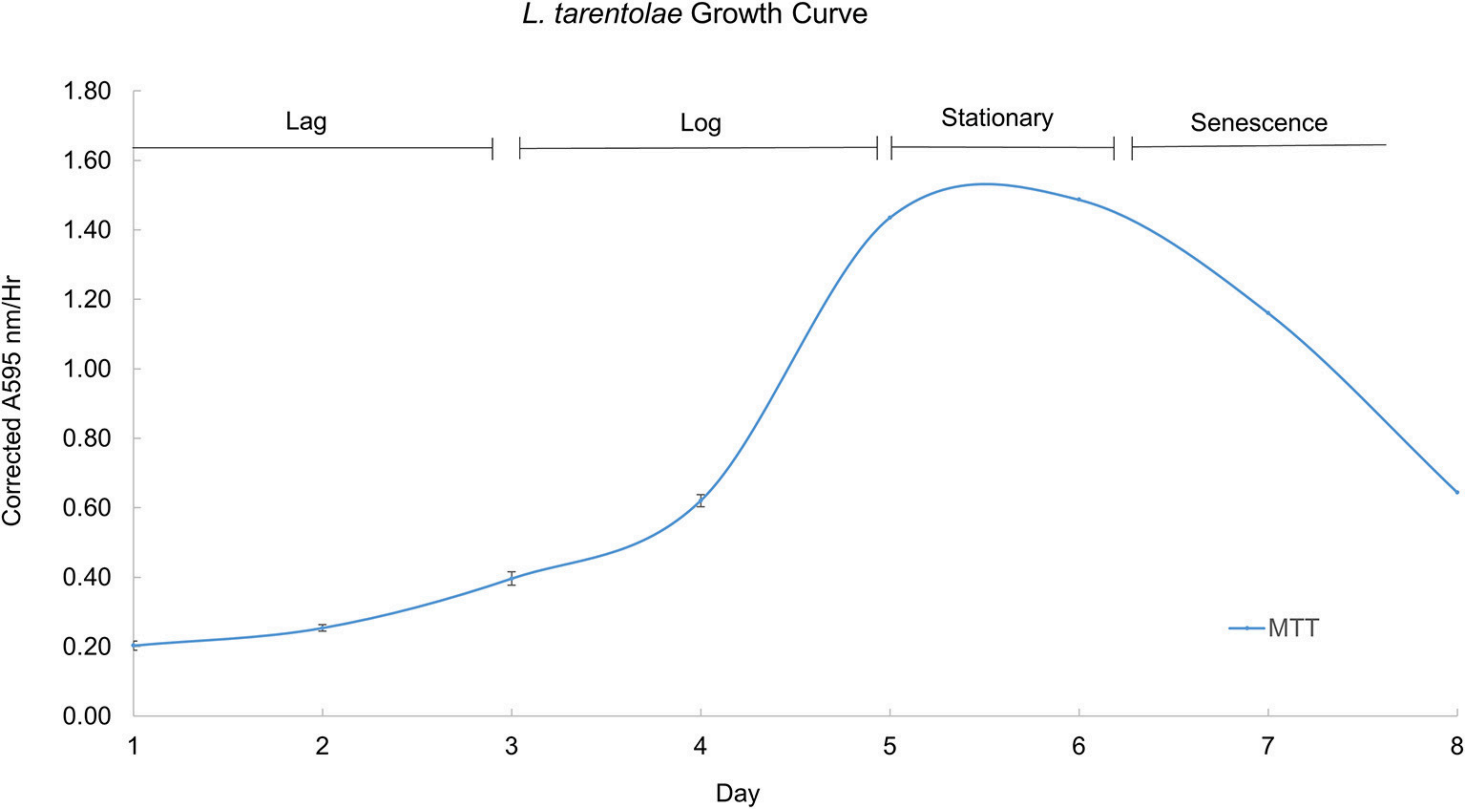
A typical growth curve for *L. tarentolae* with the corrected MTT response plotted on the Y-axis and the day in culture plotted on the X-axis. The lag phase occurs on days 1–3. The log phase occurs on days 4–5. The stationary phase occurs between days 5 and 6. The senescence phase occurs on days 6–8. Each point is the mean ± standard deviation of *n* = 4 replicates.

During their growth curve, *L. tarentolae* respond in a predictable manner to the MTT reagent. This predictable and repeatable response is useful as a metric because it gives a context for normal *L. tarentolae* behavior, and serves a reference point from which enzyme pools, whole cells or cell supernatant, are collected. Furthermore, knowing what phase of the growth curve cells are in is useful because it allows more accurate interpretation of an effective potential treatment. Figure 4 shows a typical growth curve with the four characteristic phases of *in vitro* cell growth exhibited by *L. tarentolae*.

### Secreted acid phosphatase enzyme assay

**Figure 5:**
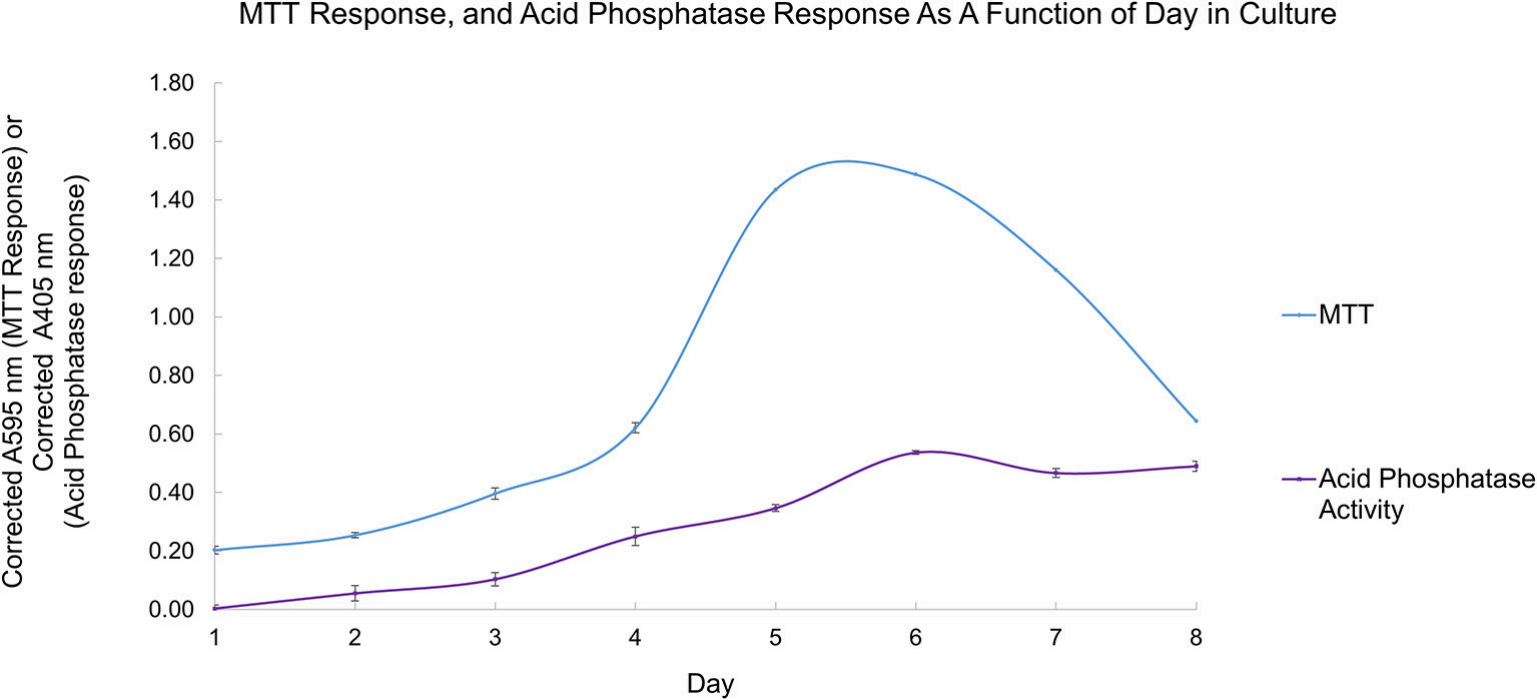
Secreted acid phosphatase activity detected as a function of day in culture (purple curve). The typical *L. tarentolae* growth curve evaluated by MTT response (blue curve). Each point is the mean ± standard deviation of *n* = 3 replicates (secreted acid phosphatase assay) or *n* = 4 (MTT response).

Using the growth curve as a reference point, detectable secreted acid phosphatase activity tracks with the MTT response up to day 6, as shown in Figure 5. When the cells' response to the MTT reagent decreases, the detectable secreted acid phosphatase activity plateaus (days 6–8). Secreted acid phosphatase activity for *L. tarentolae* supernatant is detectable on all 8 days of a typical *L. tarentolae* growth curve. Because the log phase is likely the most relevant phase to the *Leishmania* infection cycle, log phase supernatant was used for the inhibition and spectroscopic studies in this work.

### Secreted acid phosphatase enzyme inhibition assays

**Figure 6:**
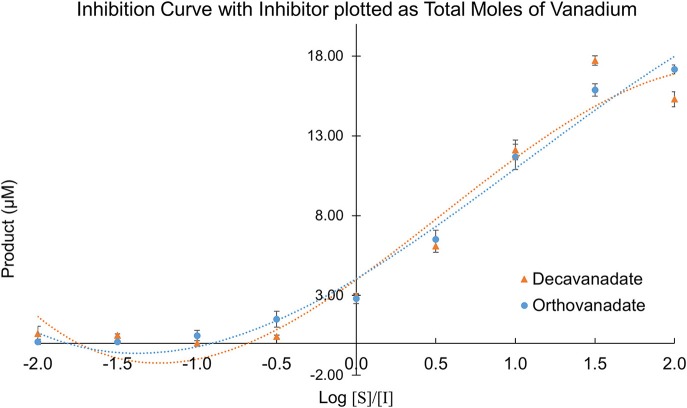
*L. tarentolae* secreted acid phosphatase enzyme activity (Y-axis) when incubated with decavanadate (orange curves) or orthovanadate (blue curves) plotted as a function of log [S]/[I] (X-axis). [I] is total molarity of vanadium. This plot assumes complete speciation of decavanadate to orthovanadate.

**Figure 7:**
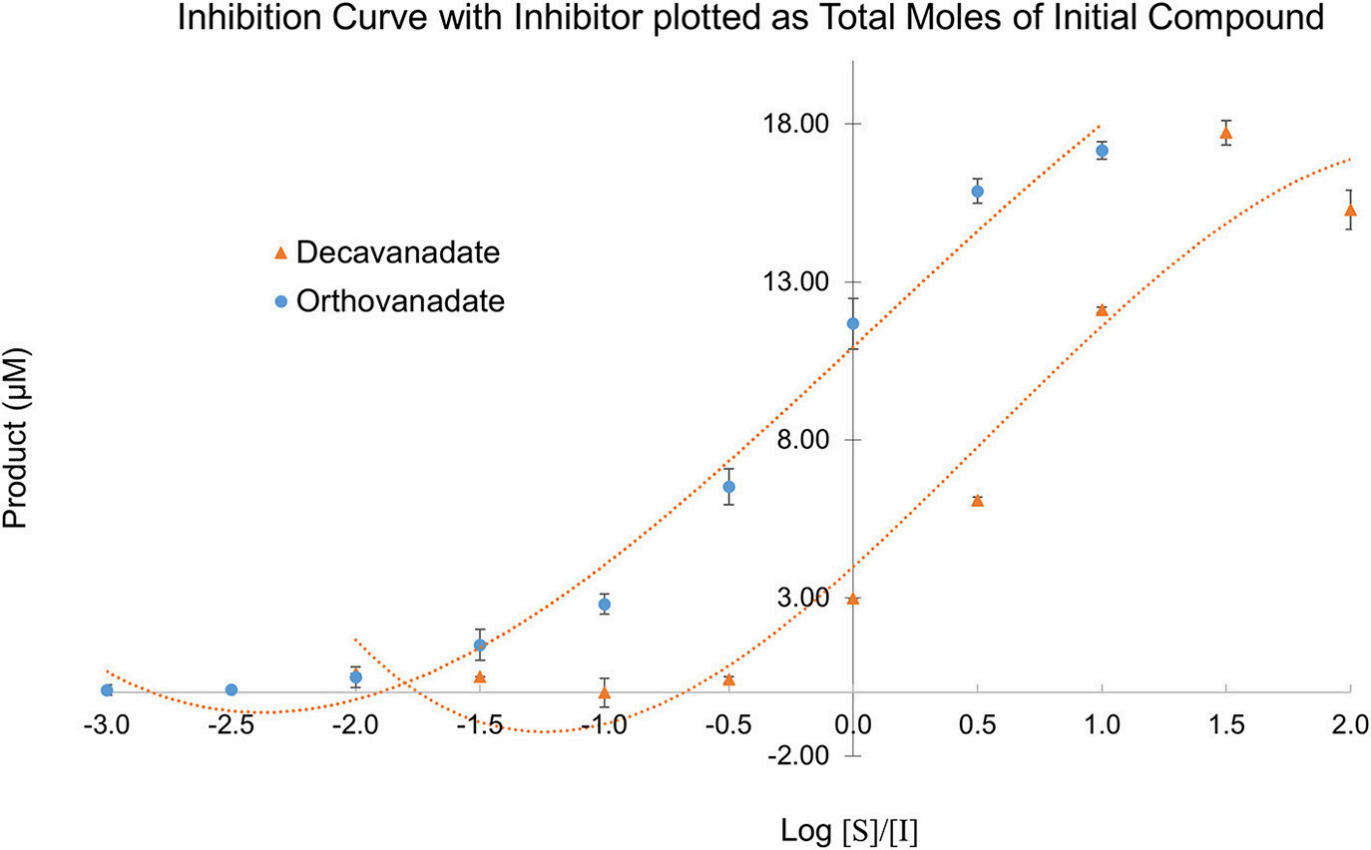
*L. tarentolae* secreted acid phosphatase enzyme activity (Y-axis) when incubated with decavanadate (orange curves) or orthovanadate (blue curves) plotted as a function of log [S]/[I] (X-axis). [I] is molarity of starting compound. This plot assumes no speciation of either decavanadate or orthovanadate occurs.

Owing to the complexity of the enzyme pool, the relative amounts of mono-, di-, tri-, tetra-, penta-, or deca-nuclear vanadium present in assay are not known. Standard speciation diagrams would indicate that under our secreted acid phosphatase assay conditions (pH = 4.5), the main form of vanadium present is decavanadate (Figure 2; Baes and Mesmer, 1976), whereas computational models typically indicate that V1 is the main species present at total vanadium concentrations in the single micromolar range. When plotting *L. tarentolae* secreted acid phosphatase enzyme activity (Y-axis) incubated with decavanadate (orange curves) or orthovanadate (blue curves) as a function of log [S]/[I] as X-axis, where [I] is either total moles of decavanadate or orthovanadate, there are three conditions where decavanadate or orthovanadate resulted in different effects from control and each other (Figure 6). These conditions are statistically significant (α = 0.05 in a paired, two-tailed *t*-test). These conditions occur when the log[S]/[I] ratio is equal to −0.5, 1.5, or 2.0. Orthovanadate is a better inhibitor in two of these conditions (log[S]/[I] = −0.5 or 2.0), and the polyoxometalate decavanadate is a better inhibitor in one condition (log [S]/[I] = 1.5). Plotting the log[S]/[I] as X-axis in this manner treats decavanadate as if it undergoes complete speciation to orthovandate. When plotting *L. tarentolae* secreted acid phosphatase enzyme activity as Y-axis, incubated with decavanadate (orange curves) or orthovanadate (blue curves), as a function of log [S]/[I] (X-axis), where [I] is total moles of starting compound, there are six conditions where decavanadate or orthovanadate resulted in different effects from control and each other (Figure 7). These conditions are statistically significant (α = 0.05 in a paired, two-tailed *t*-test). These conditions occur when the log[S]/[I] ratio is equal to −1.5, −1.0, −0.5, 0.0, 0.5, or 1.0. It should be noted that at log[S]/[I] = −3.0 and −2.5, there were no decavanadate values for product formation to compare to the orthovanadate samples, and at log[S]/[I] = 1.5 and 2.0, there are no orthovanadate values to compare to the decavanadate values.

Despite the complex equilibrium dynamics of vanadium in aqueous solutions which might lead one to predict different speciation depending on the starting conditions, it is predicted that at micromolar concentrations, decavanadate will undergo speciation completely to orthovanadate (Rehder, 2008). In such a case, we should expect to see identical experimental results for sufficiently low total concentrations of vanadium, independent of the choice of either orthovanadate or decavanadate starting material. Even with identical total moles of vanadium, though, we see differing values depending on the starting species. Decavanadate is a better inhibitor in all six of these experimental conditions. Plotting the data in this manner treats decavanadate and orthovanadate as if neither compound undergoes any speciation. Under this assumption, decavanadate is consistently better at inhibiting *L. tarentolae* secreted acid phosphatase activity on a mole of compound basis.

These plots represent the extremes of speciation: complete speciation of vanadium to overwhelmingly one form (Figure 6) or no speciation of vanadium (Figure 7), leaving either decavanadate or orthovanadate completely intact. We recognize that the accurate description of what occurs to these vanadium complexes under SAP assay conditions likely lies in between the extremes depicted. As mentioned previously, multiple factors affect oxovanadium speciation. The interpretation of these data to answer the question, which oxovanadium complex is best at inhibiting *L. tarentolae* SAP activity, is largely a function of being able to solve the speciation dilemma under our assay conditions. Currently, it is reported that decavanadate is thermodynamically unstable at micromolar concentrations and undergoes speciation almost entirely to orthovandate (Rehder, 2008). Therefore, orthovanadate was used at 10 times the molar concentration as decavanadate as a control to see the difference in impact of V_1_ vs. V_10_. The equilibria expressions describing the speciation of decavanadate to orthovandate, however, are non-linear. Thus, it does not suffice to use orthovanadate at 10 times the concentration of decavanadate. When using orthovanadate at 10 times the molar concentration as decavanadate, and comparing samples with the same total moles of vanadium, our UV-Visible spectroscopic data indicate that at multiple local maxima (A375 nm, A405 nm, A415 nm) and millimolar vanadium concentrations, the samples are different (as shown in Tables 2–**4**). We are not able to report that the samples containing single or double digit micromolar concentrations of total vanadium were different. This is largely due to our inability to detect sample absorbance values above background at these low concentrations given the nature of the milieu.

**Table 2:** Electronic absorption (UV-Visible) results at 375 nm after 24 h of incubation.

**Total vanadium (μM)**	**Molarity of starting material**	**Mean corrected A_375_ nm**	***p*-value**	**Statistically different?**
39,100	3,910	1.423	0.038	Yes
	39,100	1.440		
12,400	1,240	0.713	0.210	No
	12,400	0.719		
3,910	391	0.465	0.001	Yes
	3,910	0.261		
1,240	124	0.380	0.384	No
	1,240	0.071		
391	391	0.019	1.000	No
	391	0.019		
124	1.24	0.005	0.109	No
	124	0.015		
39.1	3.91	0.000	N/A	N/A
	39.1	0.000		
12.4	1.24	0.001	0.742	No
	12.4	0.003		
3.91	0.391	0.000	0.423	No
	3.91	0.002		

*Total vanadium concentration used in spectroscopic assays, the starting amount of either decavanadate (orange shading) or orthovanadate (no shading), the corrected mean absorbance value at a single wavelength, and the calculated p-value. The statistical threshold (α = 0.05) is used in regards to the comparison between the samples containing decavanadate starting material as compared to the samples containing orthovanadate starting material*.

## Conclusions

The UV-Visible study shows that sample types containing the same total moles of vanadium, but starting with either orthovanadate starting material or decavanadate starting material do not result in the same spectroscopic data after 24 h of incubation under assay conditions (α = 0.05, in a paired, two-tailed *t*-test). We interpret these differences as implying different amounts of the oxovanadium species responsible for the absorbance measurements at A375, A405, and A415 nm are present. Data collected at A375 nm show that samples containing 39.1 or 3.91 mM total vanadium, but using decavanadate or orthovandate starting material, are different (Table 2). Data collected at A405 nm show that samples containing 12.4, 3.91, or 1.24 mM total vanadium, but using decavanadate or orthovandate starting material, are different (Table 3). Data collected at A415 nm show that samples containing 39.1, 12.4, 3.91, or 1.24 mM total vanadium, but using decavanadate or orthovandate starting material, are different (Table 4). It is interesting to note that regardless of how one assumes the X-axis should be interpreted, i.e., how one defends “solving” the speciation problem under assay conditions, both orthovanadate and decavanadate starting material are excellent inhibitors of *L. tarentolae* secreted acid phosphatase at total vanadium concentrations of 39.1, 12.4, 3.91, and 1.24 mM. It is at these concentrations of total vanadium and pH that decavanadate is reported to be the major form of vanadium present (Rossotti and Rossotti, 1956; Baes and Mesmer, 1976). It is also at these total vanadium concentrations that the UV-visible data are statistically different. Therefore, we report that decavanadate is also an inhibitor of *L. tarentolae* secreted acid phosphatase activity. This work supports the results that other vanadium speciation studies imply, that in a complex biochemical environment, speciation must be experimentally addressed and should not be assumed. Vanadium speciation is a known source of variability, and potentially significantly important for its impact on medical efficacy and patient risk. We strongly recommend investigating vanadium speciation as part of the standard methods of assessing vanadium compounds capable of speciation as therapeutics. Even so, it seems practical from a clinical perspective, that a topical formulation using easily-synthesized decavanadate may be a robust short-term treatment option for cutaneous leishmaniasis. This treatment option is robust because the pH of the skin is acidic with an average pH of 4.7 (Lambers et al., 2006), thus a pH in which decavanadate is stable.

**Table 3:** Electronic absorption (UV-Visible) results at 405 nm after 24 h of incubation.

**Total vanadium (μM)**	**Molarity of starting material**	**Mean Corrected A_405_ nm**	***p*-value**	**Statistically different?**
39,100	3,910	1.577	0.094	No
	39,100	1.603		
12,400	1,240	0.791	0.023	Yes
	12,400	0.801		
3,910	391	0.368	0.001	Yes
	3,910	0.180		
1,240	124	0.078	0.014	Yes
	1,240	0.052		
391	391	0.015	0.742	No
	391	0.015		
124	1.24	0.006	0.160	No
	124	0.019		
39.1	3.91	0.000	0.187	No
	39.1	0.004		
12.4	1.24	0.004	0.524	No
	12.4	0.007		
3.91	0.391	0.000	0.155	No
	3.91	0.000		

*Total vanadium concentration used in spectroscopic assays, the starting amount of either decavanadate (orange shading) or orthovanadate (no shading), the corrected mean absorbance value at a single wavelength, and the calculated p-value. The statistical threshold (α = 0.05) is used in regards to the comparison between the samples containing decavanadate starting material as compared to the samples containing orthovanadate starting material*.

**Table 4:** Electronic absorption (UV-Visible) results at 415 nm after 24 h of incubation.

**Total vanadium (μM)**	**Molarity of starting material**	**Mean corrected A_415_ nm**	***p*-value**	**Statistically different?**
39,100	3,910	1.690	0.000	Yes
	39,100	1.720		
12,400	1,240	0.841	0.033	Yes
	12,400	0.856		
3,910	391	0.330	0.001	Yes
	3,910	0.155		
1,240	124	0.069	0.029	Yes
	1,240	0.046		
391	391	0.013	0.742	No
	391	0.013		
124	1.24	0.003	0.702	No
	124	0.018		
39.1	3.91	0.000	0.423	No
	39.1	0.007		
12.4	1.24	0.004	0.957	No
	12.4	0.003		
3.91	0.391	0.000	0.423	No
	3.91	0.004		

*Total vanadium concentration used in spectroscopic assays, the starting amount of either decavanadate (orange shading) or orthovanadate (no shading), the corrected mean absorbance value at a single wavelength, and the calculated p-value. The statistical threshold (α = 0.05) is used in regards to the comparison between the samples containing decavanadate starting material as compared to the samples containing orthovanadate starting material*.

## Author contributions

The initial manuscript draft was prepared by BD as part of his M.S. thesis and revised by CM and MJ. Figures were prepared by BD, except as noted. All the authors had final approval of the submitted version of the paper.

### Conflict of interest statement

The authors declare that the research was conducted in the absence of any commercial or financial relationships that could be construed as a potential conflict of interest.
